# *Drosophila melanogaster* as a model for unraveling unique molecular features of epilepsy elicited by human GABA transporter 1 variants

**DOI:** 10.3389/fnins.2022.1074427

**Published:** 2023-01-19

**Authors:** Ameya S. Kasture, Florian P. Fischer, Lisa Kunert, Melanie L. Burger, Alexander C. Burgstaller, Ali El-Kasaby, Thomas Hummel, Sonja Sucic

**Affiliations:** ^1^Institute of Pharmacology, Medical University of Vienna, Vienna, Austria; ^2^Department of Neuroscience and Developmental Biology, University of Vienna, Vienna, Austria; ^3^Department of Epileptology and Neurology, University of Aachen, Aachen, Germany

**Keywords:** *Drosophila melanogaster*, epilepsy, γ -aminobutyric acid (GABA), GABA transporter 1, protein folding and trafficking, transporter disease variants, 4-phenylbutyrate, uptake

## Abstract

Mutations in the human γ-aminobutyric acid (GABA) transporter 1 (hGAT-1) can instigate myoclonic-atonic and other generalized epilepsies in the afflicted individuals. We systematically examined fifteen hGAT-1 disease variants, all of which dramatically reduced or completely abolished GABA uptake activity. Many of these loss-of-function variants were absent from their regular site of action at the cell surface, due to protein misfolding and/or impaired trafficking machinery (as verified by confocal microscopy and de-glycosylation experiments). A modest fraction of the mutants displayed correct targeting to the plasma membrane, but nonetheless rendered the mutated proteins devoid of GABA transport, possibly due to structural alterations in the GABA binding site/translocation pathway. We here focused on a folding-deficient A288V variant. In flies, A288V reiterated its impeded expression pattern, closely mimicking the ER-retention demonstrated in transfected HEK293 cells. Functionally, A288V presented a temperature-sensitive seizure phenotype in fruit flies. We employed diverse small molecules to restore the expression and activity of folding-deficient hGAT-1 epilepsy variants, *in vitro* (in HEK293 cells) and *in vivo* (in flies). We identified three compounds (chemical and pharmacological chaperones) conferring moderate rescue capacity for several variants. Our data grant crucial new insights into: (i) the molecular basis of epilepsy in patients harboring hGAT-1 mutations, and (ii) a proof-of-principle that protein folding deficits in disease-associated hGAT-1 variants can be corrected using the pharmacochaperoning approach. Such innovative pharmaco-therapeutic prospects inspire the rational design of novel drugs for alleviating the clinical symptoms triggered by the numerous emerging pathogenic mutations in hGAT-1.

## 1. Introduction

The γ-aminobutyric acid (GABA) transporter 1 (GAT-1) is one of the main four GABA transporters in the central nervous system, where it mediates neurotransmission by rapidly clearing GABA from the synapse into neurons and astrocytes. Transport *via* GAT-1 can also operate in a reverse mode, i.e., releasing GABA into extracellular spaces. GAT-1 holds the conventional neurotransmitter transporter protein fold, with twelve transmembrane domains (TMDs) and intracellular amino and carboxyl termini. Its transport mechanism also conforms to the pivotal “alternating-access” model (Jardetzky, 1966), i.e., substrate translocation *via* the transporter is set in motion by the movement of the bundle (TMD 1, 2, 6, and 7) and the scaffold (TMD 3, 4, 8, and 9) domains. For this, GAT-1 relies on an electrochemical gradient established by chloride and sodium ions across the plasma membrane, with the proposed stoichiometry of transporting a single GABA molecule entailing co-transport of one chloride and two sodium ions (Loo et al., 2000; Kanner, 2003). GAT-1 embodies an important pharmaco-therapeutic target and loss-of-function mutations in the GAT-1 gene have latterly been associated with epilepsy, intellectual disability and autism (Goodspeed et al., 2020; Mattison et al., 2018). At this juncture, GAT-1 is not alone: many other - by now prominent - examples of diseases unequivocally linked to mutations in the SLC6 protein family, include chronic orthostatic intolerance, infantile parkinsonism/dystonia, hyperekplexia and creatine transporter deficiency in the human transporters for norepinephrine (NET, SLC6A2), dopamine (DAT, SLC6A3), glycine (GLYT-2, SLC6A5) and creatine (CRT-1, SLC6A8), respectively (Asjad et al., 2017a; El-Kasaby et al., 2019; Farr et al., 2020; Bhat et al., 2021).

According to the latest reports of the World Health Organization, active epilepsy has a prevalence of 0.4–1% in the general population, with an estimated 5 million people diagnosed each year. Handling epilepsy is often a challenging endeavor. The chief goal of treatment is preventing seizures, whilst avoiding any detrimental adverse effects. Besides, despite the availability of some two dozen anti-seizure medications, approximately one-third of patients remain resistant to any pharmacotherapy (Löscher et al., 2020). This immense and unmet need for efficacious therapeutic approaches calls for detailed investigations of the underlying molecular mechanisms of this disease, to facilitate the development of improved drugs with novel mechanisms of action. The advent of next-generation sequencing has resulted in tremendous progress in the field of epilepsy genetics (Møller et al., 2015). As portrayed in our recent review (Fischer et al., 2022), numerous mutations have been identified in the human SLC6A1 (GAT-1) gene in patient cohorts manifesting myoclonic-atonic epilepsy (MAE/myoclonic-astatic epilepsy/Doose syndrome) (Carvill et al., 2015; Johannesen et al., 2018; Mattison et al., 2018). Untypical to MAE, most patients manifested developmental delay prior to seizure onset, suggesting that GAT-1 variants may present a distinct syndrome combining MAE with abnormal development (Carvill et al., 2015). Based on the variants reported to date, approximately 10% of all cases appear to be inherited from affected parents (Johannesen et al., 2018; Mattison et al., 2018). Carvill and co-workers found that two of the four missense point mutations reported in their study (R44Q, A288V, G297R, and A334P), were inherited from the affected mothers (Carvill et al., 2015). Interestingly, R44 and A288 were found to play crucial functional roles long before their direct correlation with GAT-1-induced epilepsy: i.e. R44 is part of the inner gate (Rosenberg and Kanner, 2008; Ben-Yona and Kanner, 2013). G297 is a determinant of substrate specificity (Wein and Wanner, 2009; Skovstrup et al., 2010), rationalizing why its mutation to a substantially bulkier and positively charged arginine abolishes GABA transport in epilepsy patients harboring the G297R mutation. Palmer and co-workers reported a *de novo* mutation, C164Y, which disrupts a disulfide bridge linking cysteine residues 164 and C173, thus destabilizing the GAT-1 structure and activity (Palmer et al., 2016). Clearly, some nexus already exists between disease phenotypes and the preceding underlying structural/functional culprits - at least for a modest fraction of all known pathogenic hGAT-1 mutations. For the vast majority of them, however, these are yet to be elucidated. “Untangling” the molecular basis of disease, at the level of individual mutations, ought to warrant the development of best possible therapeutic strategies. At this point, none of the existing clinical approaches have proven entirely successful in treating hGAT-1-triggered epilepsies. Many patients respond reasonably well to the ketogenic diet, which is considered one of the best available MAE therapies today. GAT-1 variants have also been linked to other syndromes, including childhood absence epilepsy, early onset absence epilepsy, eyelid myoclonia with absences, Lennox-Gastaut syndrome, temporal lobe epilepsy and childhood epilepsy with centro-temporal spikes and mild intellectual disability (Johannesen et al., 2018). This convoluted array of clinical indications across the varied pool of pathological GAT-1 mutations renders treatment hugely challenging, and calls for a personalized approach to managing this condition.

We here explored the molecular and rescue mechanisms of epilepsy, instigated by fifteen hGAT-1 mutations. Impaired protein folding has been a common perpetrator in many SLC6 transporter pathologies, reported thus far. However, some of the folding-deficient transporters can be rescued by small molecules, known as chemical [e.g., 4-phenylbutyrate (4-PBA) and dimethyl sulfoxide] or pharmacological chaperones [i.e. specific transporter ligands, such as noribogaine for DAT or serotonin transporter (SERT)]. These compounds assist the folding of misfolded proteins and restore their expression at the cell surface (Sucic et al., 2016; Kasture et al., 2017; Asjad et al., 2017b; Freissmuth et al., 2018, El-Kasaby et al., 2019; Ng et al., 2021). Inhibitors of proteinaceous chaperones, i.e. heat shock proteins (HSPs) 70 and 90, also proved advantageous in salvaging misfolded mutants of several SLC6 transporters (Koban et al., 2015, Kasture et al., 2016; Asjad et al., 2017a). This capacity of the HSP relay is tangible; HSPs perform their monitor chores along the protein folding trajectory, and become released from their client proteins only once these achieve their proper folded states. This, in turn, primes them for the subsequent concentrative, COPII-mediated, exit from the endoplasmic reticulum (ER) compartment (Sucic et al., 2011; Sucic et al., 2013; Montgomery et al., 2014; Kasture et al., 2019). Upon inhibition of HSPs, the ensuing lenience in ER quality control mechanisms augments the ER export of newly synthesized proteins (Freissmuth et al., 2018). In such manner, small molecules can be applied to maneuver the cellular sequels of protein folding and trafficking, not only in heterologous systems (e.g., HEK293 cells), but also *in vivo* (e.g., flies). It is fair to posit that distinct variants might be responsive to specific drugs, or better still, a synergistic action of concurrent treatment with two or more particular compounds. The latter compels a systematic approach that is both cost and time efficient. Here, *Drosophila melanogaster* epitomizes an apt tool, which has perpetually proven its worth in the study of many different aspects of neurological disorders. GABA is the key fast inhibitory neurotransmitter in lower invertebrates (Lee et al., 2003). *Drosophila* express a single GABA transporter, expressed exclusively in astrocytes (Muthukumar et al., 2014), which closely resemble the morphological and electrophysiological features of human astrocytes (Macnamee et al., 2016). *Drosophila* has been a popular invertebrate organism to model seizure conditions (Parker et al., 2011; Howlett et al., 2013; Cunliffe et al., 2015; Lin et al., 2017; Saras et al., 2017; Rosch et al., 2019) and GAT regulation is known to modulate seizure-like activity in flies (Muthukumar et al., 2014; Mazaud et al., 2019). We opted for utilizing flies to probe the intricate molecular grounds behind the severe clinical manifestations of hGAT-1-induced epilepsy disorders. We envisage that our new insights may advance the impending therapeutic strategies for many young patients afflicted with this debilitating condition.

## 2. Materials and methods

### 2.1. Reagents

[^3^H]GABA (specific activity: 25–40 Ci/mmol) was obtained from PerkinElmer Life Sciences (Llantrisant, United Kingdom). Liothyronine was purchased from Tocris Bioscience (Bristol, United Kingdom). 4-Phenylbutyrate (4-PBA), liothyronine and tiagabine were obtained from Sigma-Aldrich (St. Louis, MO, USA). Cell culture media, supplements, and antibiotics were obtained from Invitrogen. SDS was from BioMol GmbH (Hamburg, Germany). Bovine serum albumin (BSA) and Complete TM protease inhibitor mixture were purchased from Roche Applied Science (Mannheim, Germany). Tris and scintillation mixture (Rotiszint^®^ eco plus) were ordered from Carl Roth GmbH (Karlsruhe, Germany). Anti-green fluorescent protein (GFP) antibodies (ab290 and A-11122) were purchased from Abcam Plc (Cambridge, UK) and Invitrogen (Life Technologies, Carlsbad, CA, United States), respectively. All other chemicals were of analytical grade. The MNCD2: hybridoma, monoclonal antibody (developed by M. Takeichi and H. Matsunami) was obtained from the Developmental Studies Hybridoma Bank, created by the NICHD of the NIH and maintained at The University of Iowa, Department of Biology (Iowa City, IA 52242, USA).

### 2.2. Methods

#### 2.2.1. Site-directed mutagenesis and cell culture

Mutations were introduced into plasmids encoding yellow fluorescent protein (YFP)-hGAT-1 using the QuikChange Lightning site-directed mutagenesis kit (Agilent Technologies, Santa Clara, CA, United States). The mutagenic primers were designed using the QuikChange primer design tool provided by the manufacturer. Human embryonic kidney (HEK293) cells were cultured in a humidified atmosphere (37^°^C and 5% CO2) in Dulbecco’s modified Eagle’s medium (DMEM) supplemented with 10% fetal calf serum and penicillin (60 mg/L) and streptomycin (100 mg/L). 24 h after seeding, the cells were transiently transfected with plasmids encoding wild-type YFP-hGAT-1 or mutants thereof, using Lipofectamine 2000 (Life Technologies, Carlsbad, CA, United States) for [^3^H]GABA uptake assays or jetPRIME ^®^ (Polyplus Transfection™) for confocal laser scanning microscopy, according to the protocols supplied by the manufacturers.

#### 2.2.2. Radioligand GABA uptake assays

Transiently transfected HEK293 cells were seeded (∼10^5^ cells per well) onto poly-D-lysine-coated 48-well plates and incubated in the absence or presence of 4-PBA (5 mM), liothyronine (100 μM) and tiagabine (10 μM). After 24 h, the medium was removed, the cells were carefully washed three times with 500 μl Krebs-HEPES buffer (10 mM HEPES, 120 mM NaCl, 3 mM KCl, 2 mM CaCl2, 2 mM MgCl2, 2 mM glucose monohydrate, pH 7.3) and subsequently incubated with 50 nM [^3^H]GABA at room temperature for exactly 3 min. Thereafter, the cells were washed with 500 μl ice-cold Krebs-HEPES buffer to terminate the uptake and subsequently lysed by adding 500 μl of 1% SDS solution. Non-specific uptake was defined in the presence of 10 μM tiagabine, with a 10 min preincubation. Quantification of [^3^H]GABA uptake was determined by liquid scintillation counting in a ß-counter.

#### 2.2.3. Confocal laser scanning microscopy and volume measurement

Confocal laser scanning microscopy was carried out to investigate the cellular localization of the wild type and mutated transporters. HEK293 cells transiently expressing YFP-tagged WT hGAT-1 or mutants thereof were seeded onto poly-D-lysine-coated ibidi^®^ glass bottom chambers. After 24 h, cell imaging was performed using a Zeiss LSM510 microscope equipped with an argon laser (at 30 milliwatts) and a 63x oil immersion objective (Zeiss Plan-Neofluar). Imaris 9.3 (Oxford instruments) was used to perform volume measurements shown in Figures 6K, L. Neuronal cadherin (NCAD) staining was used to define the antennal lobe and surfaces option was used to identify and calculate the volumes of WT hGAT-1 and the A288V variant.

#### 2.2.4. Enzymatic de-glycosylation and immunoblotting

After 48 h, transiently transfected HEK293 cells were lysed in a buffer containing 50 mM Tris.HCl (pH 8.0), 150 mM NaCl, 1% dodecylmaltoside, 1 mM EDTA, and a protease inhibitor mixture (Roche Complete™); the lysates were rotated at 4^°^C for 1 h. Subsequently, insoluble material was removed by centrifugation (30 min at 13,000 g at 4^°^C). Aliquots of the lysate (protein content 20 μg) were incubated in the absence or presence of endoglycosidase H, using the NEB assay kit, according to the instructions provided by the manufacturer. Equal amounts of protein were separated by denaturing polyacrylamide gel electrophoresis (resolving gel 7% monomer concentration) and transferred onto nitrocellulose membranes. The membranes were blocked with blocking buffer [5% skimmed milk in Tris-buffered saline containing 0.1% Tween 20 (TBST)] and then incubated with a rabbit polyclonal anti-GFP antibody (1:5000 dilution) overnight at 4^°^C. After washing, the immunoreactivity was detected by fluorescence, using a donkey anti-rabbit secondary antibody at 1:5000 dilution (IRDye 680RD, LICOR).

#### 2.2.5. *Drosophila* genetics and drug treatment

The transgenic UAS reporter lines for the wild type YFP–tagged hGAT-1 and the A288V variant were generated using the pUASg attB vector (Bischof et al., 2007) and injected into embryos of ZH-86Fb flies (Bloomington stock no. 24749). To avoid any positional effect, phi-integrase system was used to target the YFP–tagged hGAT-1 and the A288V reporter lines on the third chromosome (landing site 86Fb). All flies were kept at 25^°^C in a 12-h light/12 h dark cycle, and the crosses were performed at 25^°^C. ALRM Gal4 (Bloomington stock no. 67031), glutamic acid decarboxylase 1 (GAD-1) Gal4 (Bloomington stock no. 51630), RPT1 RNAi (Bloomington stock no. 33930), UAS-mCD8-GFP (FBti0012686), UAS-KDEL-RFP (Bloomington stock no. 30909) were ordered from the Bloomington *Drosophila* stock center (Bloomington, IN). Flies of the required genotype received standard cornmeal medium/food, supplemented with either water, 1 mM 4-PBA, 100 μM tiagabine or 100 μM liothyronine. 4-PBA and tiagabine were dissolved in water (medium) and 50 mM stock solutions of liothyronine were prepared in DMSO. Flies were fed for 48 h and were then either imaged or used for behavior studies.

#### 2.2.6. Immunohistochemistry and imaging

Adult fly brains were dissected in PBS and fixed in 2% paraformaldehyde in PBS, for 1 h at room temperature. Subsequently, the brains were washed in 0.1% Triton X-100 in PBS (PBST) three times for 20 min and blocked in 10% goat serum for 1 h at room temperature. They were next incubated in solutions containing antibodies directed against GFP (1:1000 dilution; A-11122, Invitrogen, Vienna, Austria) and neuronal cadherin (1:20, DSHB, Iowa, IA, USA), overnight in 10% goat serum at 4^°^C. Thereafter, the brains were washed three times in PBST for 20 min before incubating with Alexa Fluor 488 goat anti-rabbit IgG (1:500; Invitrogen, Vienna, Austria) and Alexa Fluor 647 goat anti-mouse IgG (1:500; Invitrogen, Vienna, Austria) secondary antibodies in 10% goat serum for 3 h at room temperature. The brains were washed three times with PBST and were mounted using Vectashield^®^ (Vector Laboratory, Burlingame, CA, United States). Images were captured on a Leica SP5II confocal microscope with 20-fold magnification. Z-stack images were scanned at 1.5 μm section intervals with a resolution of 512 × 512 pixels. Images were processed using Image J.

#### 2.2.7. Heat-induced seizure and climbing assays

Ten 3–5 days-old male flies of said genotypes were isolated in individual plastic vials using CO_2_. Flies were allowed to recover and acclimate to the new environment for 1–2 h, before the vials were immersed in a water-bath at 40^°^C for 2 min, or for a period of 1–5 min (for data shown in Figure 4A). The 40^°^C temperature point was chosen for heat induction, based on the protocols established in previous studies (Byers et al., 2021; Mituzaite et al., 2021). Seizures were defined as a period of brief leg twitches, followed by failure to maintain a standing posture, wing flapping and leg twitching. After 2 or 5 min (Figure 4A), the vials were removed from the water-bath.

For climbing assays, ten 3–5 days-old male flies of the pertinent genotypes were isolated in individual plastic vials. Additional plastic vials were taped and used to create 18 cm long climbing chambers. Flies were tapped down to the bottom of the vials and allowed to climb for 30 s. Flies that climbed 70% of the total distance (i.e., 12.6 cm) within 30 s were counted.

## 3. Results

### 3.1. Expression and function deficits of hGAT-1 variants associated with epilepsy

**FIGURE 1:**
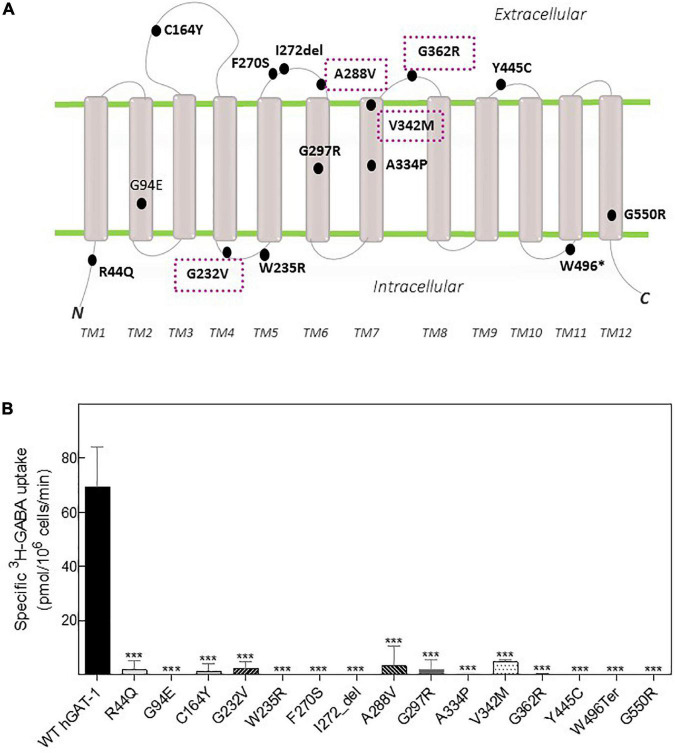
Topology model and GABA transport activity of human GABA transporter 1 (GAT-1) variants linked to epilepsy. **(A)** Epilepsy variants mapped onto the hGAT-1 topology, indicating their respective positions. Four recurrent variants are encapsulated (dotted boxes). N and C, amino and carboxyl termini, respectively; TM, transmembrane domains; “*”and “del,” termination codon and deletion mutations, respectively. **(B)** HEK293 cell were transfected with plasmids encoding yellow fluorescent protein (YFP)-tagged hGAT-1 plasmids encoding the indicated mutations and wild type hGAT-1 as control. Specific [^3^H]GABA uptake was measured for exactly 3 min, as described in “2 Materials and methods”. Non-specific uptake was determined in the presence of 10 μM tiagabine. Error bars indicate S.E.M. The data were statistically analyzed by one-way ANOVA, followed by Tukey’s *post-hoc t*-tests [***P < 0.001, compared to WT hGAT-1 (control)].

**FIGURE 2:**
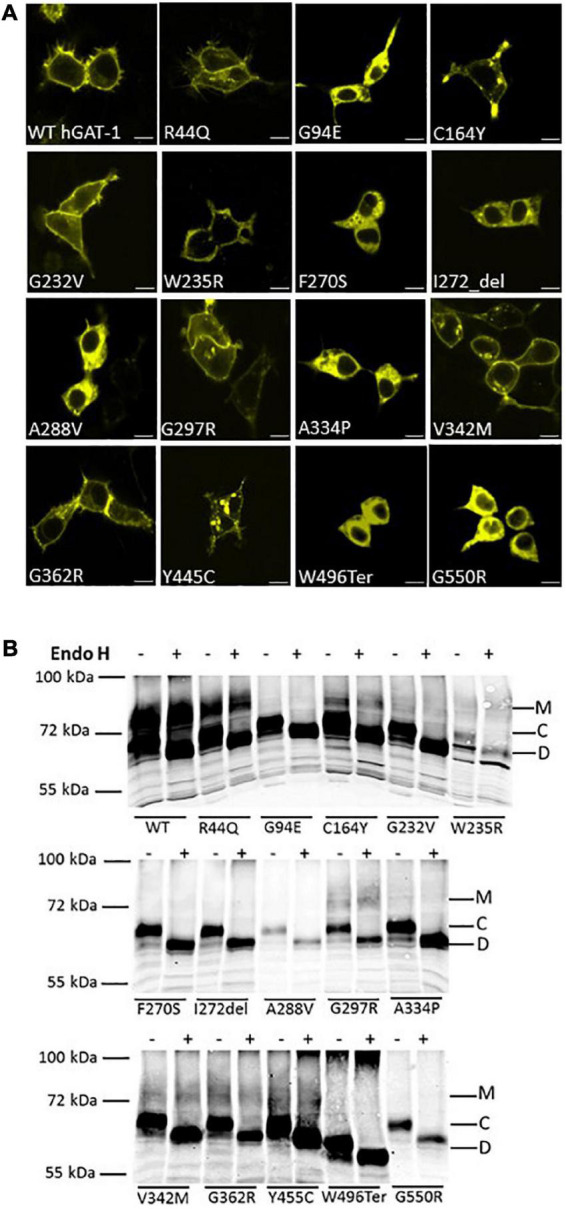
Cellular localization of wild type and disease variants of human GABA transporter 1 (GAT-1). **(A)** HEK293 cell were transfected with plasmids encoding yellow fluorescent protein (YFP)-tagged GATs to visualize their expression. HEK293 cell were transfected with plasmids encoding N-terminally YFP-tagged WT hGAT-1 and 15 epilepsy mutants thereof. Scale bars represent 10 μm. 24 h after transfection, the cells were seeded onto poly-D-lysine-coated ibidi^®^ glass bottom chambers. Confocal laser scanning microscopy was performed as described under “2 Materials and methods”. WT hGAT-1 targets to the cell surface, while many of the epilepsy variants indicate an altered (i.e., intracellular) expression patterns. **(B)** Representative blots showing de-glycosylation patterns of WT hGAT-1 and epilepsy mutants with endoglycosidase (Endo) H. Detergent lysates were prepared from HEK293 cells expressing YFP-tagged GATs. Immunoblotting was performed using an antibody against the YFP-tag. The mature glycosylated, core-glycosylated and de-glycosylated bands are designated M, C, and D.

Fifteen variants of hGAT-1 associated with epilepsy (Figure 1A), were transiently expressed in HEK293 cells. Radioligand transport assays (Figure 1B), revealed that all variants triggered functional defects, i.e., dramatically reducing or completely abolishing GABA uptake. One conceivable cause for the observed functional defects is protein misfolding, which leads to ER-retention of the affected variant transporters. We visualized the subcellular localization of all mutants in HEK293 cells using confocal laser scanning microscopy. The N-termini of all transporters were tagged with the yellow fluorescent protein (YFP) to monitor their cellular distribution. The YFP-tag did not affect GAT-1 activity (i.e., the average specific uptake values were 69.6 ± 4.8 and 73.4 ± 6.3 pmol/10^6^ cells/min for the untagged versus YFP-tagged wild type hGAT-1 constructs, respectively). The images (Figure 2A) showed that the majority of the variants accumulated within the cells (i.e., G94E, C164Y, F270S, I272_del, A288V, A334P, Y445C, W496_ter, and G550R). On the other hand, WT hGAT-1 and several mutants (R44Q, G232V, W235R, G297R, V342M, and G362R) were targeted to the plasma membrane. To verify these results, we studied the glycosylation state of each variant. ER-resident transporters are core-glycosylated and sensitive to de-glycosylation by endoglycosidase H. Transporter proteins located at the plasma membrane are mature glycosylated and resistant to endoglycosidase H. As illustrated in Figure 2B, two protein bands were detected for WT hGAT-1. The lower band was sensitive to endoglycosidase-H, which represents the core-glycosylated ER-retained species. The upper band was resistant to enzymatic digestion, corresponding to the mature glycosylated protein species. Based on these data, we infer that functional defects, exhibited by many of the variants, resulted from impaired protein folding mechanisms. However, these results do not clarify the compromised uptake function of those variants which displayed normal surface expression, indicating possible conformational defects in e.g., GABA binding and/or its translocation *via* hGAT-1.

### 3.2. *Drosophila* as a model to study the trafficking and functionality of GAT-1 variants

**FIGURE 3:**
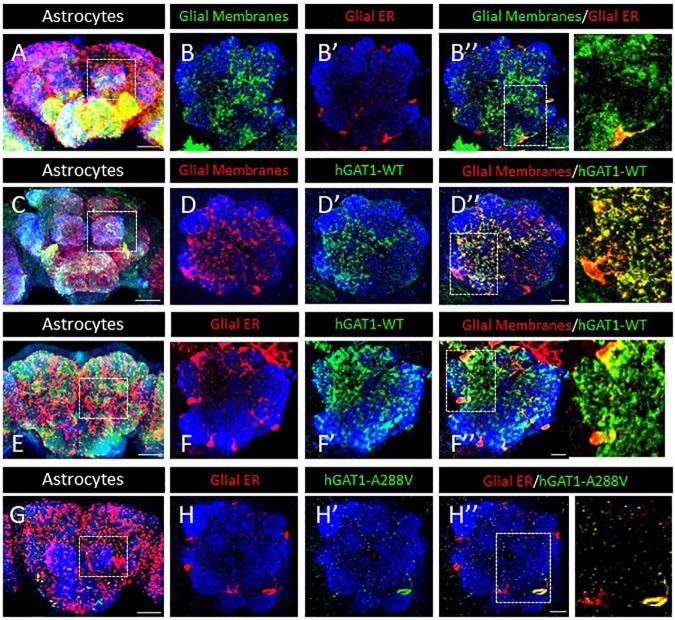
Human GABA transporter 1 (hGAT-1) A288V mutant shows ER retention in adult fly brain. **(A,B)** Organization of astrocytes in the adult *Drosophila* central brain. Cell bodies of astrocytes cover the brain surface, also around the antennal lobes (dashed line in A). **(B–B”)** Astrocyte membranes penetrate into the AL neuropile **(B)**, while the glial ER compartments are restricted to the peripheral cell bodies **(B’, B”)**. **(C,D)** Distribution of a wild type YFP-hGAT-1 transgene in astrocytes of the adult brain **(C)** and in the antennal lobes with a co-localization of wild type YFP-hGAT-1 with central neuropile glial processes **(D–D”)**. **(E,F)** Distribution of a wild type YFP-hGAT-1 transgene in astrocytes of the adult brain **(E)** and in the antennal lobes with only minor co-localization of wild type YFP-hGAT-1 with the ER compartment in peripheral glial cell bodies **(F–F”)**. **(G,H)** Distribution of a mutant YFP-hGAT-1-A288V transgene in astrocytes of the adult brain **(E)** and in the antennal lobes. No YFP-hGAT-1-A288V can be detected in most astrocytes and in rare cases of visible protein expression YFP-hGAT-1-A288V is restricted to the endoplasmic reticulum (ER) compartment **(H–H”)**. Scale bar for panel **(B,D,F,H)** is 10 μm and for panel **(A,C,E,G)** is 50 μm. Genotypes: **(A–B”)**:; *UAS-KDEL-RFP/ALRM Gal4; UAS-mCD8-GFP/* + *;* **(C–D”)**:;*ALRM Gal4/* +*; UAS-YFP-hGAT-1 WT/UAS-mCD8-GFP* (E-F”):; *UAS-KDEL-RFP/ALRM Gal4; UAS-YFP WT-hGAT-1/* +*;* and **(G–H”)**:; *UAS-KDEL-RFP/ALRM Gal4; UAS-YFP-hGAT-1-A288V/* + *;*. Each image is representative of at least 10 additional images per condition. Neuropiles are labeled by NCAD in blue.

Our prior studies on folding-deficient DAT and SERT mutants created a solid platform for examining GAT-1 (*SLC6A1)* in fruit flies (Asjad et al., 2017a; Kasture et al., 2019). We generated transgenic flies expressing fluorescently labeled WT hGAT-1 and the A288V variant thereof, in a Gal4 driver-dependent manner. *Drosophila* express the GABA transporter (dGAT) exclusively in astrocytes. Therefore, ALRM Gal4 driver line, which mainly labels astrocytes, was used to drive the expression of WT hGAT-1 and A288V. First, we visualized the ER and cell surface of astrocytes in adult fly brains (Figure 3A). Astrocytes are broadly expressed across the fly brain and we focused on the single section of the antennal lobe to better visualize the expression of the transgenes. Figure 3B–B” show membrane (B) and ER (B’) expression of the astrocytes. Next, we co-expressed the tagged WT hGAT-1 with membrane (Figures 3C, D”) and ER markers (Figures 3E, F”) in astrocytes, and found that the WT hGAT-1 expression is extensive and resembles that of the tagged membrane CD8 (Figures 3C, D”). Contrary to WT hGAT-1, the A288V mutant showed co-localization with the ER marker (Figures 3G, H”). We also analyzed the expression of WT hGAT-1 and A288V in the GABAergic neurons of adult flies, and found that the mutant resides primarily (trapped) in the cell bodies (Supplementary Figures 1A–C).

**FIGURE 4:**
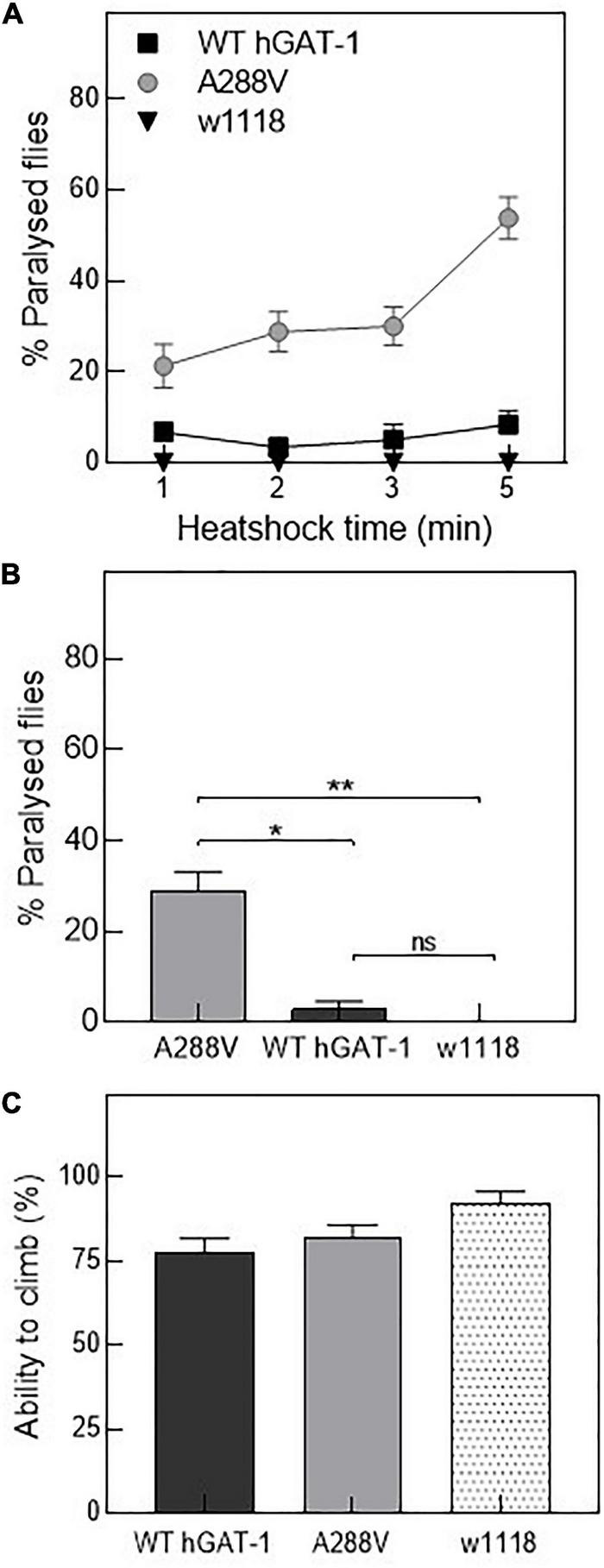
Human GABA transporter 1 (hGAT-1) A288V mutant displays a heat-induced seizure phenotype. **(A,B)** Seizure susceptibility in different genotypes as indicated by percentage of flies displaying the seizure repertoire after heat induction. **(A)** Three to five days old 10 male flies of genotype *w1118;ALRM GAL4; UAS-YFP-hGAT-1 WT;* and *ALRM-GAL4; UAS-YFP-hGAT1-A288V* were kept in a water-bath for 1, 2, 3, and 5 min and seizure activity was studied during the said period (*n*: 6). **(B)** Shows percent paralyzed flies, following 2 min heat inductions (*n*: 6). The statistical comparison was done by analysis of variance followed by Dunn’s *post-hoc* test (*p*-value for WT compared to A288V (*) = 0.0045, *p*-value for w1118 compared to A288V (**) = 0.0003). **(C)** Climbing activity of flies was studied by assessing the ability of flies to climb 70% of 18 cm long climbing chamber in 30 s. No significant difference was observed. Means ± S.E.M. are indicated, from at least six independent experiments.

*Drosophila* has been employed to study epilepsy, and especially seizures resulting from disease-linked mutations in transporters and ion channels (Parker et al., 2011; Talsness et al., 2020; Jeong et al., 2021). Thus, we reasoned that hGAT-1 epilepsy variants ought to exhibit seizure-like activity in flies. First, we tested mechanosensitivity of the A288V variant expressed in astrocytes, using mechanical stress (i.e., by vortexing/the “bang assay”). No seizure phenotype was observed after a 10 s vortexing period in transgenic flies expressing A288V. Subsequently, we examined whether A288V is heat- or temperature-sensitive (Figures 4A, B). 3–5 days old male flies expressing A288V were transferred to plastic vials and immersed in a water-bath maintained at 40^°^C (Figure 4A). Flies exhibited increased locomotion, followed by the inability to maintain posture, wing flapping and leg twitching. The seizure repertoire was visible 30 s after immersion in the water bath. We also observed that approximately 30% of the flies showed seizure susceptibility after a 2 min immersion in water (*p* = 0.0003, Figures 4A, B) and some 50% after a 5 min time-point (Figure 4A). The flies showed variable seizure recovery periods (lasting from a few seconds to 15 min), upon removal from the water bath. Interestingly, flies expressing the WT hGAT-1 also demonstrated seizure susceptibility to some extent, which was considerably lower than that of A288V (Figures 4A, B). We also observed seizure sensibility in transgenic flies expressing only UAS A288V and UAS WT-hGAT-1 reporter lines, indicating a “leaky expression” of this transgene (Supplementary Figure 1D). Further, the climbing activity of flies expressing WT-hGAT1 and A288V was studied to assess any possible locomotor defects (Figure 4C), but our data failed to show any obvious climbing defects in these flies.

### 3.3. Rescue of hGAT-1 mutants by small molecules

**FIGURE 5:**
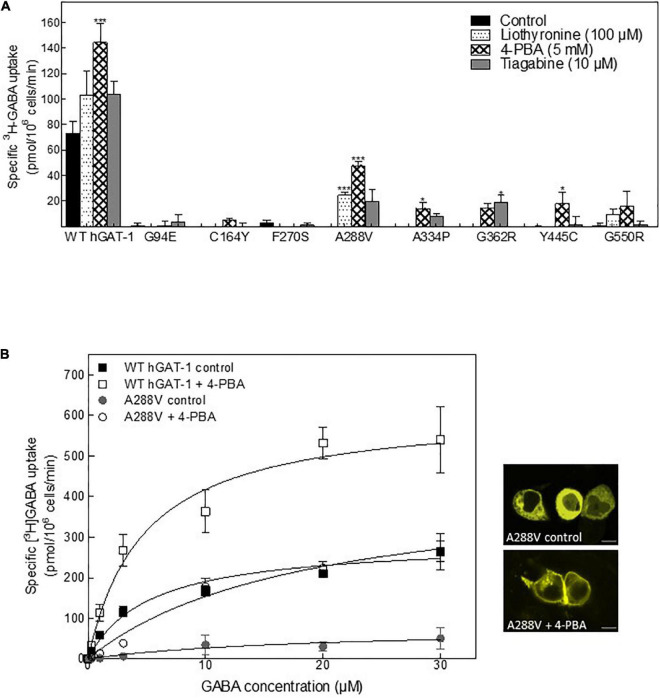
Rescue of pathogenic human GABA transporter 1 (hGAT-1) variants by pharmacochaperoning. **(A)** HEK293 cells were transiently transfected with plasmids driving the expression of yellow fluorescent protein (YFP)-tagged WT hGAT-1 and epilepsy mutants thereof. After 24 h, the cells were seeded onto 48-well plates and treated with the indicated drugs [5 mM 4-phenylbutyrate (4-PBA), 100 μM liothyronine and 10 μM tiagabine] for a subsequent 24 h. The cells were extensively washed, to remove the inhibitors (liothyronine and tiagabine), prior to measuring specific [^3^H]GABA uptake (3 min). **(B)** Michaelis-Menten [^3^H]GABA uptake kinetics for WT hGAT-1 and the A288V variant, before and after 5 mM 4-PBA treatment, according to the procedure described under **(A)**. Right-hand panels show representative confocal miscroscopy images of HEK293 cells expressing A288V, under control and 4-PBA-treated conditions (scale bar = 10 μm). The uptake data were obtained from at least three independent experiments (error bars = S.E.M.; The data were statistically compared by one-way ANOVA, followed by Tukey’s *post-hoc t*-tests (*p < 0.05, ***p < 0.001).

Folding defects can be corrected by pharmacological and chemical chaperones, thus recovering the expression and activity in the affected variant transporters (Sucic et al., 2016; Kasture et al., 2017). Previous studies showed that the HSP70 inhibitor pifithrin-μ and the HSP90 inhibitor 17-DMAG could rescue misfolded mutants of SERT and DAT (El-Kasaby et al., 2014; Kasture et al., 2016; Asjad et al., 2017a; Asjad et al., 2017b). We tested whether these compounds would also prove effective in the rescue of hGAT-1 variants. In addition, we assessed the efficacy of two novel HSP70 and HSP90 inhibitors (i.e., YM-08 and NVP-HSP990, respectively). Regrettably, none of these compounds resulted in any appreciable improvement in GABA uptake activity by any of the examined disease mutants (Supplementary Figure 2A). The chemical chaperone (and putative HSP70 modulator) 4-PBA, restored the activity of folding-deficient hCRT-1 mutants linked to severe intellectual disability and epilepsy in our recent studies (El-Kasaby et al., 2019; Farr et al., 2020). Similarly, 4-PBA proved most successful in rescuing a number of disease variants of hGAT-1 (Figure 5A), particularly the A288V mutation (Figure 5B), which displayed better surface expression (Figure 5B, right hand panel images) and saturable GABA uptake, following a 24 h exposure to 5 mM 4-PBA in HEK293 cells (the kinetic parameters of GABA uptake are shown in Table 1). This response was reaffirmed for the A288V equivalent synthetic SERT mutant A329V (Supplementary Figure 2B). Besides, we tested the pharmacochaperoning potential of GAT-1 ligands, liothyronine (Jurik et al., 2015) and the renowned antiepileptic tiagabine. Upon treatment with liothyronine, we observed a robust increase in GABA uptake by A288V (Figure 5A). Based on the fact that A288V was absent from the plasma membrane (i.e., trapped in the ER compartment, Figures 2B, 5B) before treatment, it is feasible to deduce that liothyronine may act as a pharmacological chaperone. Treatments with tiagabine (Figure 5A) restored—at least modestly- GABA uptake by another ER-retained variant A334P (Figure 5A).

**FIGURE 6:**
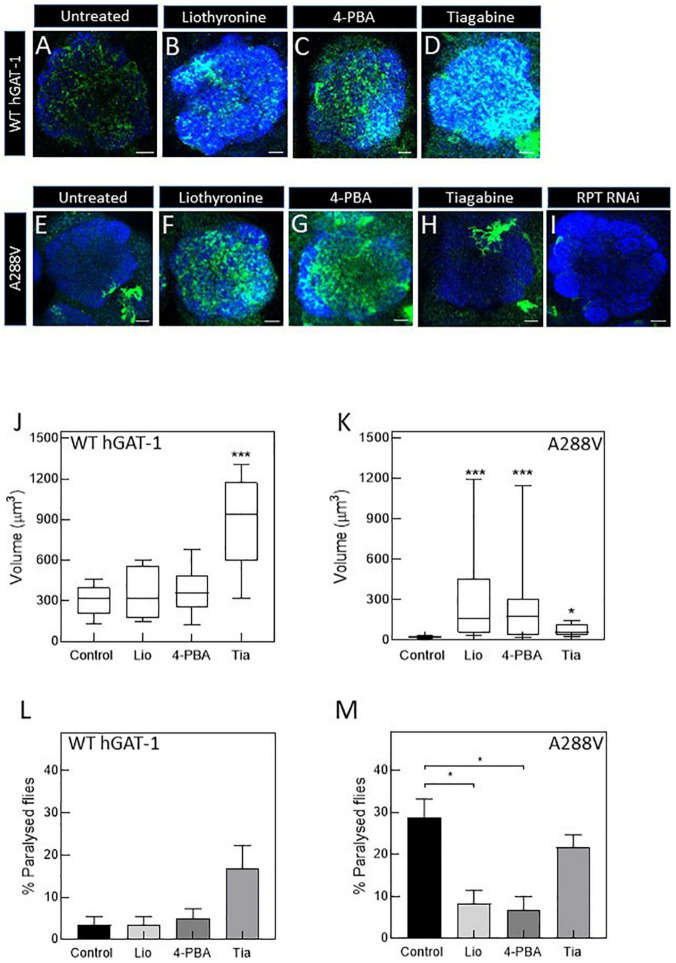
Pharmacological treatment restores the expression and activity of the A288V mutant in fruit flies. Panel **(A–D)** show astrocytic expression of WT human GABA transporter 1 (hGAT-1) in the single section of the antennal lobe of untreated **(A)**, 100 μM liothyronine **(B)**, 1 mM 4-phenylbutyrate (4-PBA) **(C)**, and 100 μM tiagabine **(D)** treated flies (Genotype:;*ALRM-GAL4; UAS-YFP-hGAT-1 WT;*). Panel **(E–H)** show astrocytic expression of hGAT-1 A288V in the single section of the antennal lobe of untreated **(E)**, 100 μM liothyronine **(F)**, 1 mM 4-PBA **(G)**, and 100 μM tiagabine **(H)** treated flies (Genotype:;*ALRM-GAL4; UAS-YFP-hGAT-1 WT;*). **(I)** Proteasomal degradation was studied using RPT1-RNAi (genotype;*ALRM-GAL4/* + *; UAS-YFP-hGAT1-A288V, UAS-RPT1;*). **(J,K)**, Expression of the WT hGAT-1 and A288V in the antennal lobe of untreated and treated flies, respectively. The expression of the transporter was quantified using Imaris 9.3 in 10 adult brains of each condition. The statistical comparison was done by analysis of variance followed by Dunn’s *post-hoc* test (*n*:10, *p < 0.05, ***p < 0.001). WT hGAT-1 and A288 flies received the food supplemented with drug for 48 h and were either dissected and imaged or were used to study seizure susceptibility [**(L,M)**, respectively]. Means ± S.E.M. are indicated from at least six independent experiments performed with 10 flies per condition. The statistical comparison was done by analysis of variance followed by Dunn’s *post-hoc* test (*p < 0.05, significantly different from control). Scale bar = 10 μm. Each image is representative of at least 10 additional images per condition. Brain neuropiles are labeled in blue by NCAD.

**TABLE 1:** Kinetic parameters of GABA uptake by wild type human GABA transporter 1 (hGAT-1) and its epilepsy variant A288V, before and after treatment with the chemical chaperone 4-phenylbutyrate (4-PBA).

	Untreated (control)		4-PBA (5 mM) treated	
	**V_max_** **(pmol.10^–6^cells.min^–1^)**	**K_m_** **(μM)**	**V_max_** **(pmol.10^–6^cells.min^–1^)**	**K_m_** **(μM)**
WT hGAT-1	289.9 ± 29.4	5.2 ± 1.7	615.7 ± 59.5	4.8 ± 1.6
A288V	n.d.	n.d.	438.6 ± 54.8	18.5 ± 4.6

K_m_ and V_max_ values were determined from data shown in Figure 5B. The values are means ± S.E.M. from three independent experiments performed in triplicate. “n.d.”, not determinable, i.e., GABA uptake activity by A288V could not be accurately measured in the absence of 4-phenylbutyrate (4-PBA) treatment. The *K_m_* values of WT and mutant transporters differed significantly with or without pharmacochaperone treatment (*p* < 0.05, Kruskal–Wallis test followed by Dunn’s post-hoc comparison).

To study the rescue efficacy of these compounds *in vivo*, we assessed whether (i) pharmacological treatment can recover the expression of A288V in adult astrocytes and (ii) reduce or abolish the seizure phenotype in fruit flies. First, we studied the effect of pharmacological treatment on the expression of the WT hGAT-1 in the antennal lobe (Figures 6A–D). We observed a widespread distribution of WT hGAT-1 in untreated (Figure 6A), liothyronine (Figure 6B), 4-PBA (Figure 6C) and tiagabine (Figure 6D) treated flies. In order to understand whether the treatment affected the expression of WT hGAT-1, its volume was measured in the entire antennal lobe, labelled with NCAD, using the Imaris 9.3 software. We observed that tiagabine treatment significantly increased the WT hGAT-1 volume in the antennal lobe (Figure 6J). Liothyronine and 4-PBA treatment did not markedly affect WT hGAT-1 volume. Next, we tested the A288V variant using the same approach, which showed very low expression in the antennal lobe (Figure 6E). A notable 12-, 9-, and 3-fold increases in the expression were observed after liothyronine (Figures 6F, K), 4-PBA (Figures 6G, K) and tiagabine treatments (Figures 6H, K), respectively. In addition, we checked whether A288V becomes rapidly cleared from astrocytes, by depleting RPT1 (a proteasome subunit) in astrocytes (Low et al., 2013). No obvious change in the expression of A288V was observed. This indicated that, although misfolded, the A288V protein is not readily targeted by the proteasome machinery (Figure 6I). Finally, we studied the effect of pharmacological treatment on the seizure phenotype. Here, liothyronine and 4-PBA treatment did not affect the seizure phenotype of WT hGAT-1 flies (Figure 6L). However, albeit not statistically significant, there was an increase in tiagabine-treated flies. The seizure phenotype of A288V flies was reduced by 71 and 75%, after liothyronine and 4-PBA treatments, respectively (Figure 6M). The tiagabine treatment had no significant effect on A288V.

## 4. Discussion

Loss-of-function disease mutations in hGAT-1 are presumed to encompass a range of physiological repercussions on GABAergic homeostasis, which is in part regulated by neuronal and glial GABA transporters. Extracellular GABA clearance is altered owing to the absence of functional hGAT-1 proteins at the synaptic cleft. Consequently, extrasynaptic GABA concentrations rise and exert effects on extrasynaptically located GABA type A and B receptors, thus prompting tonic inhibition. Presynaptic GABA pools concurrently decline, which in turn disrupts subsequent phasic neurotransmission reviewed in Fischer et al. (2022). A large fraction of the reported epilepsy-associated hGAT-1 mutations give rise to misfolded/dysfunctional transporters. Scores of pathogenic variants in other SLC6 family members, such as DAT, NET, GLYT-2, and CRT-1, were previously shown to disrupt their protein folding machinery. The clinical consequences of such mutations are typically very severe, e.g., childhood parkinsonism, intellectual disability or orthostatic intolerance, to name just a few (Bhat et al., 2021). Nonetheless, folding defects can be corrected by means of pharmacological and/or chemical chaperone. For instance, small molecules targeting the HSP relay (i.e., blockers of HSP 70 and 90) commendably rescued folding-deficient versions of DAT, SERT and CRT-1 in our previous studies (El-Kasaby et al., 2014; Kasture et al., 2016, Asjad et al., 2017a; El-Kasaby et al., 2019). We hence tested their chaperoning predisposition in reinstating GABA transport activity in epilepsy variants of hGAT-1. Contrary to our expectations, none of these molecules (i.e., pifithrin-μ, 17-DMAG, YM-08, and NVP-HSP990) proved efficacious.

The chemical chaperone 4-PBA has proven beneficial in various disease models (Kang et al., 2002; Lim et al., 2004; Qi et al., 2004; Ryu et al., 2005; Vilatoba et al., 2005; Kubota et al., 2006; Özcan et al., 2006; Datta et al., 2007; De Almeida et al., 2007; Inden et al., 2007; Ozcan et al., 2009; Debattisti et al., 2014; El-Kasaby et al., 2019; Tao et al., 2022). In our hands, 4-PBA treatment rescued several disease-linked misfolded hCRT-1 mutants associated with intellectual disability and epilepsy (El-Kasaby et al., 2019). We found that it also recuperated a number of hGAT-1 epilepsy variants in heterologous cells. To have a more clear understanding of the mechanistic facets behind the folding trajectory of hGAT-1, and owing to its scarce pharmacology, we generated a human serotonin transporter (hSERT) equivalent mutation of A288V-hGAT-1 (i.e., A329V-hSERT). Unlike the hallmark synthetic folding-deficient hSERT mutant PG-AA (El-Kasaby et al., 2014; Koban et al., 2015), A329V was not amenable to rescue by the renowned pharmacochaperone noribogaine (Supplementary Figure 2B). But, matching its hGAT-1 counterpart, A329V too showed a comparably marked response to 4-PBA. The chemical chaperone action of 4-PBA (counteracting the aggregation of unfolded proteins by binding to their hydrophobic domains) is already highly acknowledged. 4-PBA is also known to modulate (i.e., inhibit) HSP70 activity, possibly by downregulating HSC70 (Rubenstein and Zeitlin, 2000; Rubenstein and Lyons, 2001; Goldfarb et al., 2006; Suaud et al., 2011; Chanoux et al., 2013). It also acts on several other proteins (e.g., histone deacetylases) engaged in the unfolded protein response system (Konsoula and Barile, 2012). Our findings from pifithrin-μ and 4-PBA-treated cells imply that heat shock proteins HSP70 and HSC70 may exert differential effects on the folding trajectory constituents of hGAT-1. We have previously proposed the use of 4-PBA as a prospective therapeutic option for treating creatine transporter deficiency (CTD), with effective plasma concentrations (≥1 mM 4-PBA) achievable by daily drug administration of ≤20 g for adults and 0.5 g/kg for children under 20 kg (El-Kasaby et al., 2019). Almost three decades ago, 4-PBA was approved by Food and Drugs Administration (FDA) for managing other conditions in people [e.g., life-long treatment of urea cycle disorders or hyperammonemia (Iannitti and Palmieri, 2011)]. Although the FDA-approval might expedite the repurposing of 4-PBA, vigilant pediatrician guidance and further research (to determine the proper use and dosage regimen) is advised for its off-label use in CTD and hGAT-1-associated epilepsies in children. Ideally, hGAT-1-selective ligands signify the safest therapeutic agents, since these act exclusively on the “target” protein. Alas, in contrast to the opulent pharmacology of monoamine transporters (SERT, NET, and DAT), that of GAT-1 is fairly destitute, ominously limiting the pool of compounds to tap into while searching for such classical pharmacological chaperones. Beside the well-known antiepileptic drug tiagabine, the thyroid hormone liothyronine (T3), was shown to inhibit GAT-1 (Mason et al., 1987; Jurik et al., 2015). We found that both drugs hold at least some pharmacochaperone potential. To the best of our knowledge, no other pharmacological chaperones of GAT-1 have been reported in the literature to date. Treatment with liothyronine partially restored GABA uptake activity of a single variant, i.e., A288V. Tiagabine treatment alike, only modestly enhanced GABA transport by the A334P variant. Recovery of GABA uptake by these drugs alludes to their capacity to facilitate the delivery of ER-trapped GATs to the plasma membrane.

Our data provide a rationale for translating current insights from cell culture into *in vivo* studies. The fruit fly *Drosophila melanogaster* has emerged as an appealing model organism in exploring misfolded SLC6 transporter variants (Kasture et al., 2016; Asjad et al., 2017a). Fly experiments afford certain practical features, such as: (i) their lavish and sophisticated genetic toolbox, (ii) time-efficient drug screening and, lastly, (iii) their cost merits (Martin and Krantz, 2014; Kasture et al., 2018; Fischer et al., 2022). Evidently, *Drosophila melanogaster* is an epitome model for testing extensive collections of pharmacological and chemical chaperones *in vivo. Drosophila* has remained a worthy model organism in studying seizures and epilepsy. Although flies typically have no seizure phenotypes, these can be induced upon mechanical stress, heat, pharmacological treatment or electrical stimulation. Seizure susceptibility varies among different fly genotypes. Notably, the sequence similarity between human GAT-1 and *Drosophila* GAT is 38.6%. Of the pathogenic variants investigated here, the following hGAT-1 amino acid residues happen to be conserved in the fly GAT: C164, F270, A288, G297, G362, Y445, and G550. Given that GAT-1 variants are directly linked to epilepsy in people, we argued that humanized flies harboring the pertinent mutations ought to manifest seizures. As predicted, this was the case for the A288V variant, in which we observed temperature-induced seizures, possibly indicative of a dominant negative phenotype. The expression of A288V significantly improved after treatment with 4-PBA and liothyronine, and to a lesser extent by tiagabine, demonstrating sound pharmacochaperoning propensity of these drugs *in vivo*. More importantly, these results complied with behavioral data, i.e., diminished seizure activity upon 4-PBA and liothyronine exposure. This is insightful because 4-PBA was found to extend the lifespan of fruit flies (Kang et al., 2002; Debattisti et al., 2014; Tao et al., 2022). In the case of tiagabine, despite enhanced expression levels, we observed a minor, but statistically insignificant, inclination toward a reduced seizure phenotype in A288V expressing flies. Moreover, tiagabine showed a trend of higher seizure susceptibility in flies expressing the WT hGAT-1. This observation was unsurprising since tiagabine, which is effective against focal seizures, has been known to induce absence seizures (Knake et al., 1999). In addition, although to a low extent, we noted seizures in the UAS reporter line without Gal4, suggestive of a “leaky expression” of the transgene. The wild type hGAT-1 displayed some seizure activity, but to a much lesser extent than the A288V variant. To overcome these shortcomings, better molecular and genetic approaches can be employed in future studies, i.e., replacing the endogenous *GAT* gene with a human equivalent and/or expressing the transgene in a conditional manner (e.g., in the FRT-FLP system), without the UAS reporter. Creating disease-relevant mutations in *Drosophila GAT* may provide additional informative aspects of hGAT-1-linked epilepsy.

Generating large libraries of small molecules is essential to the discovery of optimal pharmacochaperone candidates for (specific/individual) disease variants in hGAT-1. Some of the drugs reported in this study, for instance, can serve as adequate lead compounds. Tiagabine is a clinically used antiepileptic already, but liothyronine is, as such, still inapt, being a synthetic form of the thyroid hormone triiodothyronine. Nonetheless, these drugs could serve as a backbone for developing additional pharmacological agents with improved therapeutic profiles. This is conceivable, given the recent progress by Gati and colleagues, who published the first crystal structure of hGAT-1 in a tiagabine-bound, inward-facing state (Motiwala et al., 2022). The availability of such data unlocks many new avenues for the rational drug design of - not only pharmacochaperone molecules - but also other important drugs, e.g., novel antiepileptics (Kanner and Dayan-Alon, 2022). Although our data indicate that pharmacochaperone action is unlikely to restore mutant uptake to wild type levels, it is known that even modest functional rescue *in vitro* can be translated into ample clinical responses [e.g., in the case of the paradigmatic folding disease, cystic fibrosis (Ramalho et al., 2002)]. We reached analogous conclusions in our studies of parkinsonism-triggering misfolded DAT variants, where miniscule rescue in dopamine uptake by HEK293 cells corresponded to a staggering recovery of the sleepless phenotype in flies (Kasture et al., 2016). Moreover, in rodent thalamus, GAT-1 is expressed exclusively in astrocytes, and the absence seizure phenotype, which is manifested in GAT-1 knockout mice, is amendable by thalamic expression of GAT-1 (Cope et al., 2009). This further highlights the significance of our findings, that *in vivo* restoration of functional glial GAT is crucial in GAT-1-associated epilepsies. Lastly, our current work shows that disease variants of hGAT-1 are responsive to pharmacochaperoning and commends the use of flies in deciphering the molecular underpinnings of transporter pathologies, and efficient drug screening of vast chemical libraries.

## Data availability statement

The original contributions presented in this study are included in the article/Supplementary material, further inquiries can be directed to the corresponding author.

## Author contributions

ASK, TH, and SS conceptualized, designed, and analyzed the experiments. ACB, ASK, AE-K, LK, MLB, FPF, and SS performed experiments. ASK, FPF, and SS wrote the manuscript. All authors contributed to the article and approved the submitted version.

## Supplementary material

Supplementary Figure 1 Expression of WT human GABA transporter 1 (hGAT-1) and A288V variant in the GABAergic neurons and seizure susceptibility of reporter and driver lines. Panels **(A–C)** show confocal images of the anterior half of adult fly brain. Glutamic acid decarboxylase 1 (GAD-1) Gal4 driver line is used to express the proteins in GABAergic neurons. Left panel shows membrane [**(A)**,; *UAS mCD8-GFP*,*GAD1 Gal4*], WT hGAT-1 [**(B)**,; *GAD1 Gal4/* + *; UAS-YFP-WT-hGAT1/* +], A288V [**(C)**,; *GAD1 Gal4/* + *,; UAS-YFP-hGAT1-A288V/* + *;*] and right panel shows respective merged images with neuronal cadherin (NCAD). (Scale bar = 50 μm). Note that the WT hGAT-1 labels the mushroom body lobes whereas, the A288V mutant remains retained in the cell body, which contains the ER. **(D)** Heat induced seizure activity of UAS reporter and Gal4 driver line. Seizure susceptibility in flies with genotype; *UAS-YFP-hGAT-1 WT;,; UAS-YFP-hGAT1-A288V;,;ALRM Gal4;* and *w1118* was studied by immersing the vials containing 10 flies of said genotype in 40^°^C water-bath for 2 min. The statistical comparison was done by analysis of variance followed by Dunn’s *post-hoc* test (*p*-value for WT compared to A288V (*) = 0.0423, *p*-value for w1118 and ALRM Gal4 compared to A288V (**) = 0.0061). Means ± S.E.M. are indicated from at least six independent experiments.

Supplementary Figure 2 Treatment of wild type and two folding-deficient epilepsy variants of hGAT-1 with heat shock protein (HSP) inhibitors, and of wild type hSERT and its A329V mutant with 4-phenylbutyrate (4-PBA) and noribogaine. **(A)** HEK293 cells were transiently transfected with plasmids encoding YFP-tagged WT human GABA transporter 1 (hGAT-1) and two disease variants thereof (A288V and A334P). A day after transfection, the cells were seeded onto 48-well plates and treated (for 24 h) with the pertinent drugs (i.e., HSP 70 inhibitors pifithrin-μ (5 μM) and YM-08 (6 μM), as well as HSP90 inhibitors 17-dimethylaminoethylamino-17-demethoxygeldanamycin (17-DMAG; 2 μM) and NVP-HSP990 (100 nM). NVP-HSP990 was ordered from Selleck Chemicals (Houston, Tx, USA). Pifithrin-μ, 17-DMAG and YM-08 were obtained from Sigma-Aldrich (St. Louis, MO, USA). The cells were carefully washed and specific [^3^H]GABA uptake (3 min) determined, as described in the “2 Materials and methods” section. The data were obtained from at least three independent experiments (error bars = S.E.M.); The data were statistically compared by one-way ANOVA, followed by Tukey’s *post-hoc t*-tests, and revealed no significant differences among the treatments, for any of the examined transporters (ns, *p* > 0.05). **(B)** HEK293 cells were transiently transfected with plasmids encoding YFP-tagged WT hSERT and A329V, a synthetic mutant equivalent to the hGAT-1 epilepsy variant A288V. 24 h after transfection, the cells were seeded onto 48-well plates and treated with the indicated drugs (5 mM 4-PBA or 10 μM noribogaine) for a subsequent 24 h. The cells were extensively washed, to remove the drugs, prior to measuring specific [^3^H]5-HT (specific activity: 41.3 Ci/mmol) uptake for exactly 1 min (non-specific uptake was determined in the presence of 10 μM paroxetine). The data were obtained from at least three independent experiments (error bars = S.E.M.). The data were statistically compared by one-way ANOVA, followed by Tukey’s *post-hoc t*-tests (**p* < 0.05).
