# Supplementary figures and images for: Drosophila melanogaster as a model for unraveling unique molecular features of epilepsy elicited by human GABA transporter 1 variants
Source: Front Neurosci. 2023 Jan 19;16:1074427. doi: 10.3389/fnins.2022.1074427 (PMC9893286; doi:10.3389/fnins.2022.1074427)

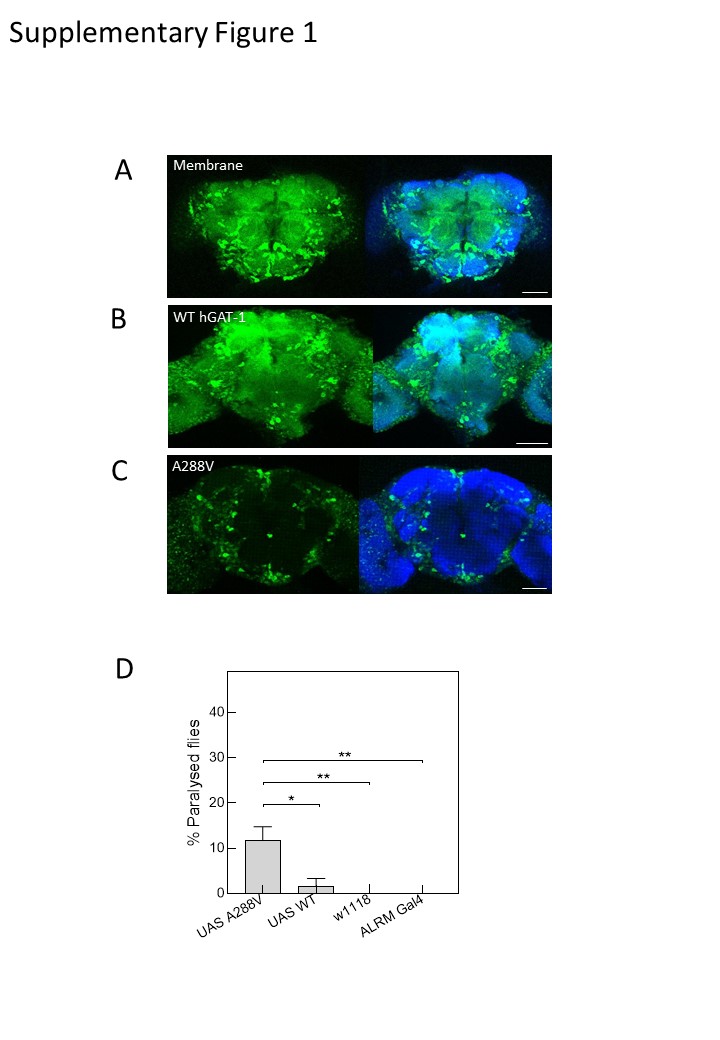

Supplement: Supplementary Figure 1 — Expression of WT human GABA transporter 1 (hGAT-1) and A288V variant in the GABAergic neurons and seizure susceptibility of reporter and driver lines. Panels (A–C) show confocal images of the anterior half of adult fly brain. Glutamic acid decarboxylase 1 (GAD-1) Gal4 driver line is used to express the proteins in GABAergic neurons. Left panel shows membrane [(A),; UAS mCD8-GFP,GAD1 Gal4], WT hGAT-1 [(B),; GAD1 Gal4/ + ; UAS-YFP-WT-hGAT1/ +], A288V [(C),; GAD1 Gal4/ + ,; UAS-YFP-hGAT1-A288V/ + ;] and right panel shows respective merged images with neuronal cadherin (NCAD). (Scale bar = 50 μm). Note that the WT hGAT-1 labels the mushroom body lobes whereas, the A288V mutant remains retained in the cell body, which contains the ER. (D) Heat induced seizure activity of UAS reporter and Gal4 driver line. Seizure susceptibility in flies with genotype; UAS-YFP-hGAT-1 WT;,; UAS-YFP-hGAT1-A288V;,;ALRM Gal4; and w1118 was studied by immersing the vials containing 10 flies of said genotype in 40°C water-bath for 2 min. The statistical comparison was done by analysis of variance followed by Dunn’s post-hoc test (p-value for WT compared to A288V (*) = 0.0423, p-value for w1118 and ALRM Gal4 compared to A288V (**) = 0.0061). Means ± S.E.M. are indicated from at least six independent experiments. [file Image_1.jpeg]

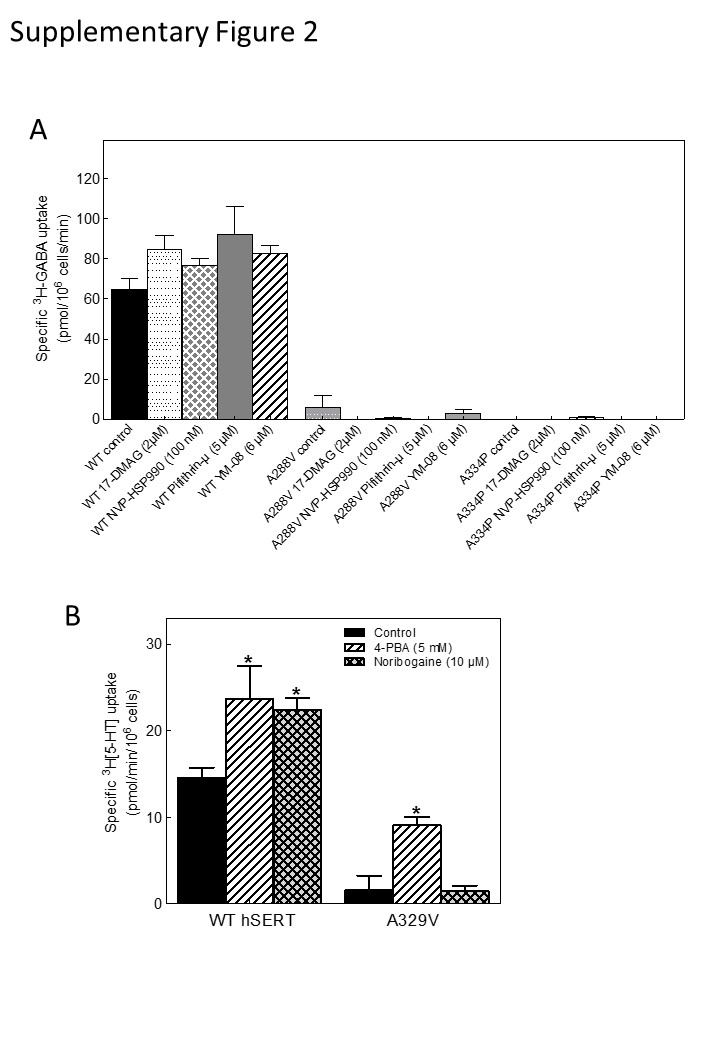

Supplement: Supplementary Figure 2 — Treatment of wild type and two folding-deficient epilepsy variants of hGAT-1 with heat shock protein (HSP) inhibitors, and of wild type hSERT and its A329V mutant with 4-phenylbutyrate (4-PBA) and noribogaine. (A) HEK293 cells were transiently transfected with plasmids encoding YFP-tagged WT human GABA transporter 1 (hGAT-1) and two disease variants thereof (A288V and A334P). A day after transfection, the cells were seeded onto 48-well plates and treated (for 24 h) with the pertinent drugs (i.e., HSP 70 inhibitors pifithrin-μ (5 μM) and YM-08 (6 μM), as well as HSP90 inhibitors 17-dimethylaminoethylamino-17-demethoxygeldanamycin (17-DMAG; 2 μM) and NVP-HSP990 (100 nM). NVP-HSP990 was ordered from Selleck Chemicals (Houston, Tx, USA). Pifithrin-μ, 17-DMAG and YM-08 were obtained from Sigma-Aldrich (St. Louis, MO, USA). The cells were carefully washed and specific [3H]GABA uptake (3 min) determined, as described in the “2 Materials and methods” section. The data were obtained from at least three independent experiments (error bars = S.E.M.); The data were statistically compared by one-way ANOVA, followed by Tukey’s post-hoc t-tests, and revealed no significant differences among the treatments, for any of the examined transporters (ns, p > 0.05). (B) HEK293 cells were transiently transfected with plasmids encoding YFP-tagged WT hSERT and A329V, a synthetic mutant equivalent to the hGAT-1 epilepsy variant A288V. 24 h after transfection, the cells were seeded onto 48-well plates and treated with the indicated drugs (5 mM 4-PBA or 10 μM noribogaine) for a subsequent 24 h. The cells were extensively washed, to remove the drugs, prior to measuring specific [3H]5-HT (specific activity: 41.3 Ci/mmol) uptake for exactly 1 min (non-specific uptake was determined in the presence of 10 μM paroxetine). The data were obtained from at least three independent experiments (error bars = S.E.M.). The data were statistically compared by one-way ANOVA, followed by Tukey’s post-hoc t-tests [file Image_2.jpeg]
